# Progress in the Study of Non-Coding RNAs in Multidifferentiation Potential of Dental-Derived Mesenchymal Stem Cells

**DOI:** 10.3389/fgene.2022.854285

**Published:** 2022-04-05

**Authors:** Biyun Zeng, Junhui Huang

**Affiliations:** Department of Oral Pathology, Xiangya Stomatological Hospital & Hunan Key Laboratory of Oral Health Research & Hunan 3D Printing Engineering Research Center of Oral Care, Central South University, Changsha, China

**Keywords:** dental-derived mesenchymal stem cells, non-coding RNAs, multi-directional differentiation, osteogenic differentiation, odontogenic differentiation

## Abstract

For decades, the desire for tissue regeneration has never been quenched. Dental-derived mesenchymal stem cells (DMSCs), with the potential of self-renewal and multi-directional differentiation, have attracted much attention in this topic. Growing evidence suggests that non-coding RNAs (ncRNAs) can activate various regulatory processes. Even with a slight decrease or increase in expression, ncRNAs can weaken or even subvert cellular fate. Therefore, a systematic interpretation of ncRNAs that guide the differentiation of DMSCs into cells of other tissue types is urgently needed. In this review, we introduce the roles of ncRNAs in the differentiation of DMSCs, such as osteogenic differentiation, odontogenic differentiation, neurogenic differentiation, angiogenic differentiation and myogenic differentiation. Additionally, we illustrate the regulatory mechanisms of ncRNAs in the differentiation of DMSCs, such as epigenetic regulation, transcriptional regulation, mRNA modulation, miRNA sponges and signalling. Finally, we summarize the types and mechanisms of ncRNAs in the differentiation of DMSCs, such as let-7 family, miR-17∼92 family, miR-21, lncRNA H19, lncRNA ANCR, lncRNA MEG3, circRNA CDR1as and CircRNA SIPA1L1. If revealing the intricate relationship between ncRNAs and pluripotency of DMSCs 1 day, the application of DMSCs in regenerative medicine and tissue engineering will be improved. Our work could be an important stepping stone towards this future.

## Introduction

DMSCs have been widely studied because of their ready availability, easy accessibility and lack of complex ethical issues. Recently, numerous types of DMSCs have been isolated and characterized, including dental pulp stem cells (DPSCs), periodontal ligament stem cells (PDLSCs), stem cells from exfoliated deciduous teeth (SHEDs), dental follicle stem cells (DFPCs), stem cells from apical papilla (SCAPs) and gingival mesenchymal stem cells (GMSCs) (Yin et al., 2021). Similar to other stem cells, DMSCs have multiple differentiation potential and can differentiate into a variety of tissue-like cells under specific induction conditions.

NcRNAs are an abundant class of RNAs that do not encode proteins. Only a few years ago these transcripts were considered “dark matter” in the genome, but now they play leading roles in the regulation of biological processes (Cai et al., 2021). There are many types of ncRNAs, and the main classes of functional ncRNAs that are not translated into proteins include microRNA (miRNA), long ncRNA (lncRNA) and circular RNA (circRNA). MiRNAs are small (∼22 nucleotides), single-stranded, non-coding RNAs encoded by endogenous genes. MiRNA biogenesis often begins with primary miRNA (pri-miRNAs) in the nucleus. Following processing by the Drosha and DRCG8, precursor miRNA (pre-miRNA) is exported from the nucleus by exportin 5. Then, it undergoes further processing to generate mature miRNA. LncRNAs are large non-coding RNAs >200 nucleotides in length, transcribed by RNA polymerase II, and its biogenesis process is similar to miRNA. CircRNAs differ from linear RNAs in that they can form covalently closed continuous loops with their 3′ heads and 5′ tails bound together and their unique closed-loop structure makes them more resistant to RNAseR digestion (Sangere et al., 1976; Danan et al., 2012). At the same time, it has highly conserved characteristics and specificity in expression patterns between cells, tissues and developmental stages (Mumtaz et al., 2020). In the present review, we summarize the important role of miRNA, lncRNA and circRNA in the multidirectional differentiation of DMSCs.

## ncRNAs and the Differentiation of DMSCs

**FIGURE 1:**
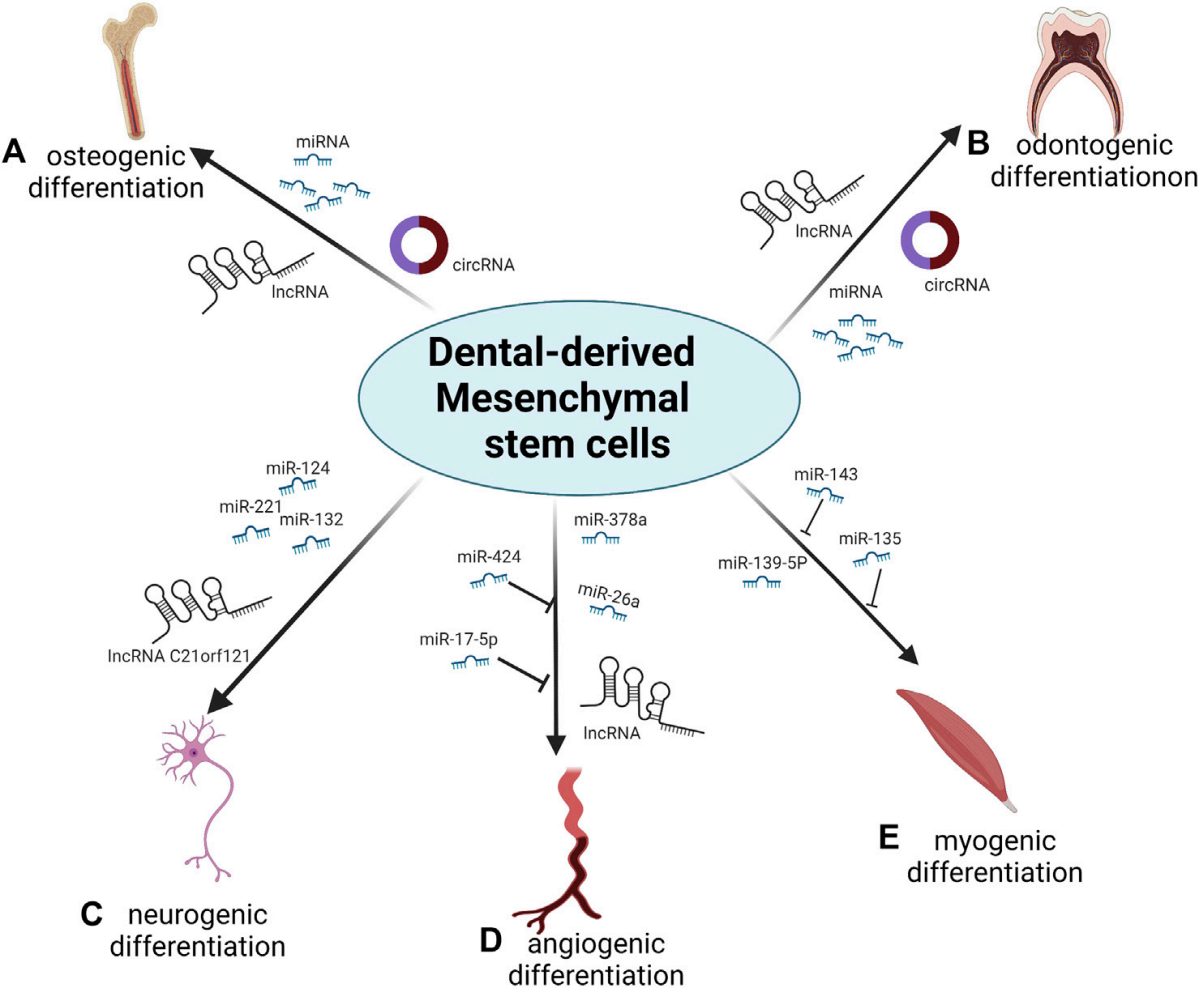
Overview of the role of ncRNAs during differentiation of DMSCs. **(A)** Numerous miRNAs, lncRNAs and circRNAs regulate the osteogenic differentiation of DMSCs. **(B)** Numerous miRNAs, lncRNAs and circRNAs regulate the odontogenic differentiation of DMSCs. **(C)** miR-132, miR-124, miR-221and lncRNA C21orf121 promote the neurogenic differentiation of DMSCs. **(D)** miR-378a and miR-26a promote the angiogenic differentiation of DMSCs; miR-424 and miR-17-5p inhibit the angiogenic differentiation of DMSCs; numerous lncRNAs regulate the angiogenic differentiation of DMSCs. **(E)** miR-139-5p promotes the myogenic differentiation of DMSCs; miR-143 and miR-135 inhibit the myogenic differentiation of DMSCs.

Many miRNAs, lncRNAs and circRNAs are associated with the differentiation of DMSCs (Figure 1). NcRNAs provide an additional and promising possibility of the regulation of the differentiation of DMSCs that has not been fully elucidated to date. With increasing numbers of ncRNAs discovered in this process, it has become possible to use these ncRNA-related therapeutic methods in the field of regenerative medicine and tissue engineering.

### ncRNAs and the Osteogenic Differentiation of DMSCs

Hao used (Hao et al., 2017) a miRNA microarray to detect the different expression profiles of miRNAs in PDLSCs during the osteogenic differentiation process. The results indicated a significant change in the expression level of 116 miRNAs(30 upregulated and 86 downregulated) in PDLSCs after 14d of osteogenic induction. (Zheng et al., 2018) used RNA sequencing to detect the different expression profiles of lncRNAs in PDLSCs at different time points during osteogenic differentiation. The results showed that 48 lncRNAs had significant changes on days 3, 7 and 14, of which 17 lncRNAs were upregulated and 31 were downregulated in PDLSCs. (Gu et al., 2017) compared the lncRNA profiles and circRNAs profiles of PDLSCs on the seventh day with or without osteogenic differentiation medium using RNA sequencing. The results showed that 17 lncRNAs were upregulated and 31 were downregulated while 766 circRNAs were significantly upregulated and 690 circRNAs were downregulated during osteogenic differentiation in PDLSCs. These results suggest an important regulatory role that ncRNAs might play in the osteogenic differentiation of PDLSCs.

### ncRNAs and the Odontogenic Differentiation of DMSCs

A microarray analysis revealed that (Gong et al., 2012) 12 upregulated miRNAs and 10 downregulated miRNAs to be differentially expressed in human DPSCs during odontogenic differentiation process. Further bioinformatic analysis showed that the target genes of these miRNAs are related to the mitogen-activated protein kinase (MAPK) and the Wnt signalling pathways; both pathways are of particular interest to odontogenesis. (Zheng and Jia, 2016) used RNA sequencing to compare the lncRNA profiles between freshly-isolated and cultured dental mesenchymal cells. The analysis indicated that there were a total of 144 lncRNAs (among which 108 were upregulated and 36 were downregulated) that participated in odontogenic differentiation. They also constructed 54 coexpression relationships in the odontogenic process, as well as an lncRNA-mRNA coexpression network. There are 139 differentially expressed genes between odontogenic induced and undifferentiated human DPSCs, with downstream pathways implicating cell cycle, extracellular matrix receptor interaction, and transforming growth factor (TGF)-β signaling pathways (Chen et al., 2016). Microarray research conducted by Chen et al. (2020) showed that 187 circRNAs are differentially expressed after odontogenic induction of human DPSCs. Moreover, hsa_circRNA_104101 was highly assumed to enhance the odontogenic differentiation of human DPSCs. These results indicate that ncRNAs might play crucial roles in this process and regulate odontogenesis-related pathways.

### ncRNAs and the Neurogenic Differentiation of DMSCs

Retinal diseases are the leading cause of irreversible visual impairment and blindness in developed countries. In the process of transdifferentiation of hPDLSCs into retinal ganglion-like cells, 71 human miRNAs were differentially expressed (44 were up-regulated and 27 were down-regulated), among which miR-132 was a key regulator of retinal fate determination of hPDLSCs (Ng et al., 2015). In the past, auditory neuropathy (loss or degeneration of spiral ganglion neurons [SGN]) was considered a serious problem with no alternative therapy. MiR-124 was a key regulator of neuronal development in the inner ear and was expressed at high levels in auditory and vestibular neurons. Some scholars transfected hDPSCs with miR-124 to observe the changes of neural progenitor cells and protein levels, and found that the expression of neural marker Nestin could be changed, which opened up broad prospects for the treatment and replacement of SGN (Mehri-Ghahfarrokhi et al., 2019). Chromatin unwinding DNA-binding protein 8(CHD8) inhibited the onset of autism spectrum disorder (ASD), and the upregulation of miR-221 activated the Wnt/β-catenin pathway by binding to CHD8, which facilitating the differentiation from SHEDs to neurons (Wen et al., 2020). Autism is a childhood neurodevelopmental disorder with complex genetic origin. A study in a rat model of autism found that lncRNA C21orf121 promotes the differentiation of SHEDs into neurons by targeting miR-140-5p to upregulate BMP2 (Liu J. et al., 2018).

### ncRNAs and the Angiogenic Differentiation of DMSCs

Growing evidence reveals that miRNAs play a critical role in angiogenic processes (Hernández-Romero et al., 2019). When cultured in a medium supplemented with bFGF and VEGF-165, DPSCs were induced toward endothelial differentiation, during which miR-424 was downregulated gradually, revealing that miR-424 negatively regulated the endothelial differentiation of DPSCs (Liu et al., 2014). Extracellular vesicles secreted by periodontitis-compromised DPSCs (P-EVs) carrying miR-378a promoted endothelial cells angiogenesis by downregulating Sufu to activate the Hedgehog/Gli1 signalling pathway (Zhou et al., 2021). In addition, overexpression of miR-17-5p blocked the pro-angiogenic ability of inflamed PDLSCs (Zhang Z. et al., 2020). SHED aggregate exosomes shuttled miR-26a promote angiogenesis both in SHED and Human umbilical vein endothelial cells (HUVECs) via TGF-β/SMAD2/3 signalling (Wu et al., 2021). An lncRNA microarray analysis showed that (Li X. et al., 2020) 376 lncRNAs were significantly increased and 426 lncRNAs were down-regulated among DPSCs cultured in vascular induction medium, suggesting that lncRNAs were also essential for angiogenic processes.

### ncRNAs and the Myogenic Differentiation of DMSCs

MiRNAs also play a crucial role in the induction of myogenic differentiation. MiR-139-5p elevated the skeletal myogenic differentiation of hDPSCs by interacting with the Wnt/β-catenin signaling pathway (Xie and Shen, 2018). The expression of miR-143 and miR-135 were significantly downregulated in 5-Aza-CdR-induced myoblast DPSCs. Interestingly, the addition of miR-143 or miR-135 inhibitors to the culture medium stimulates the myogenic properties of DPSCs, which eventually fuses to form myotube (Li et al., 2015).

## Regulatory Mechanisms of ncRNAs in the Differentiation of DMSCs

**FIGURE 2:**
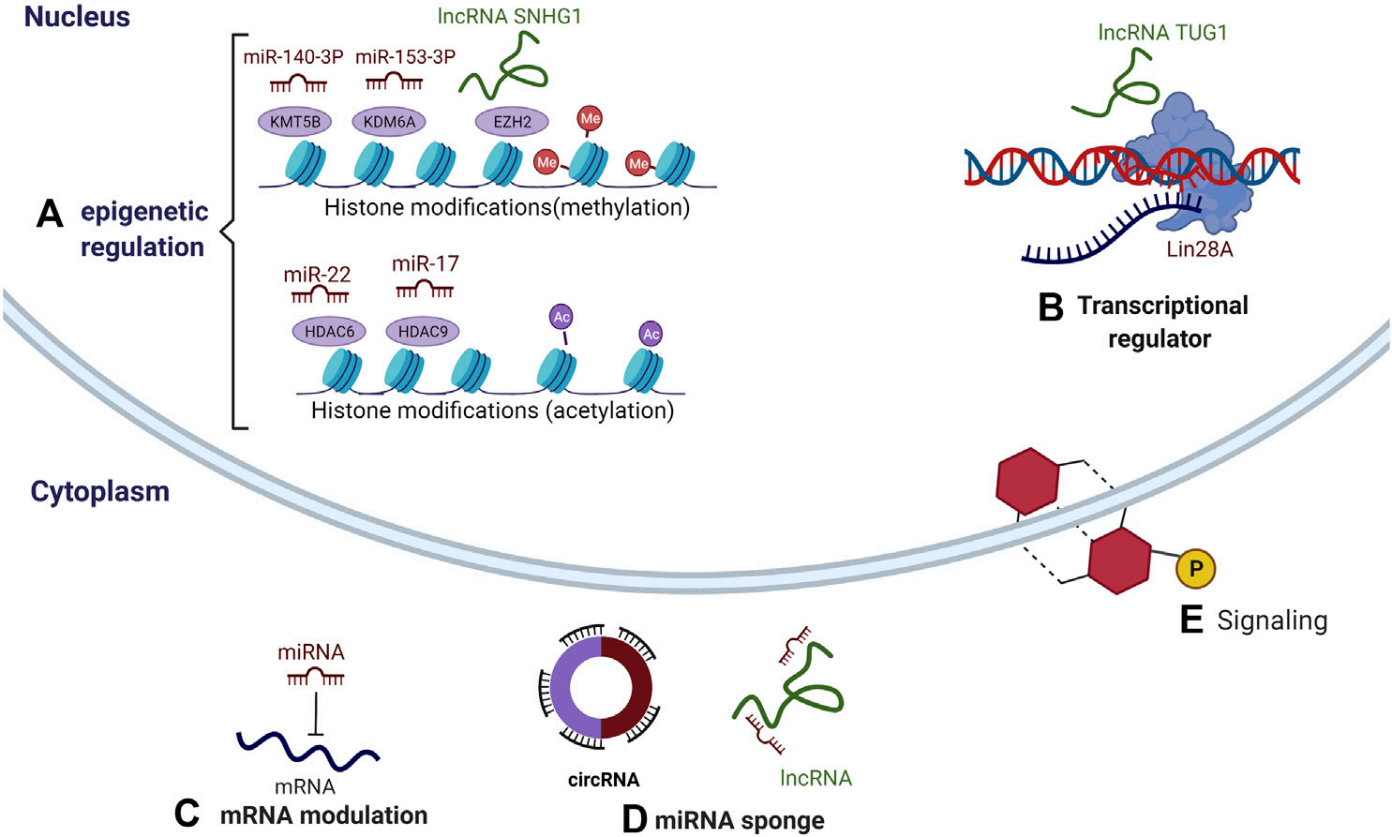
Multiple mechanisms of ncRNAs regulating the differentiation of DMSCs. **(A)** Epigenetic regulation: miR-140-3p interacts with KMT5B, miR-153-3p interacts with KDM6A, and lncRNA SNHG1 interacts with EZH2, resulting in abnormal methylation. miR-22 interacts with HDAC6, and miR-17 interacts with HDAC9, resulting in abnormal acetylation. **(B)** gene transcription: lncRNA TUG1 interacts with RBP family member-Lin28A, affecting the transcriptional activation of downstream genes. **(C)** mRNA modulation: miRNA targets the 3′ untranslated region (UTR) of mRNA. **(D)** miRNA sponges: lncRNAs and circular RNAs target miRNAs. **(E)** signaling: all three ncRNAs can participate in signaling pathways.

ncRNAs can regulate the differentiation of DMSCs by diverse mechanisms (Figure 2).

### Epigenetic Regulation

Common epigenetic regulation mainly involves multifaceted dynamics, including DNA methylation, histone modifications. It has been reported that mature miRNAs presence in the nucleus, which can activate or silence genes through mechanisms of epigenetic pathways. MiRNAs involved in epigenetic modifications are referred to as epi-miRNAs, such as miR-140-3p, miR-153-3p, miR-22, miR-17. Lysine methyltransferase 5B(KMT5B) belongs to a group of histone methyltransferases (Rodas-Junco et al., 2017; Shen et al., 2019). (Zheng et al., 2021) found that miR-140-3p enhances the osteo/odontogenic differentiation of DPSCs by inhibiting KMT5B under the hypoxia condition simulated by cobalt chloride (CoCl2). Lysine demethylase 6A (KDM6A) belongs to a group of histone demethylases. (Jiang and Jia, 2021) revealed that miR-153-3p inhibits osteogenic differentiation of PDLSCs through KDM6A-induced demethylation of H3K27me3 (trimethylated histone H3 Lysine 27). HDACs can transfer acetyl groups from the histone, resulting in histone hypoacetylation and chromatin accumulation. MiR-22 promoted osteogenic differentiation of human PDLSCs by targeting HDAC6 (Yan et al., 2017). (Li L. et al., 2018) discovered that miR-17 and HDAC9 formed an inhibitory loop under chronic inflammatory conditions, which negatively affected each other. There were HDAC9-enriched promoter regions in miR-17, while miR-17 induced PDLSCS osteogenesis by inhibiting HDAC9.

In the nucleus, lncRNAs regulate the gene expression in various ways. These include epigenetic modifications by the recruitment of chromatin modifiers. Enhancer of the zeste homolog 2 (EZH2) is a histone methyltransferase, specifically in charge of methylation of H3K27me3. IncRNA SNHG1 inhibited the osteogenic differentiation of PDLSCs through EZH2-mediated H3K27me3 methylation of Kruppel-like factor 2(KLF2) promotor (Li X. et al., 2020).

### Transcriptional Regulation

LncRNAs can specifically bind with a specific family of proteins called RNA binding proteins (RBPs) to regulate the biological functions of these proteins and then affect the transcriptional activation of downstream genes. As a novel protein type, RBPs have been demonstrated to interact with lncRNAs and have attracted increasing attention from researchers (Kim et al., 2017; Kishore et al., 2011; Wu et al., 2016). He et al. (2018) demonstrated that lin-28 homologue A (Lin28A, a member of the RBP family) contains multiple binding sites of lncRNA-taurine upregulated gene 1 (TUG1) and lncRNA-TUG1 facilitates osteogenic differentiation of PDLSCs via interacting with Lin28A.

### mRNA Modulation

In the cytoplasm, binding of mature miRNAs to the 3′ untranslated region (UTR) of specific mRNA targets leads to gene repression by undermining mRNA stability or reducing translation. For example, miRNAs negatively regulate the osteogenesis of DMSCs by targeting the 3′-UTR of osteogenic genes, such as alkaline phosphatase (ALP), osteocalcin (OCN), Runt-related transcription factor 2 (RUNX2), etc. MiR-584-5p inhibits the osteogenic differentiation of human PDLCs by binding to ALPL mRNA and reducing the expression of ALPL protein (Wang et al., 2021). MiR-218 has been confirmed to target Runx2 and play important inhibitory roles in the osteogenic differentiation of PDLSCs, DPSCs and GSCs (Gay et al., 2014). MiR-204 negatively regulates the osteogenic differentiation of human DFPCs (hDPCs) by targeting Runx2 and ALP (Ito et al., 2020).

### miRNA Sponges

Some lncRNAs and circular RNAs regulate the activity of miRNAs because they have binding sites that retain them, thus modulating the activity of miRNAs. LncRNAs and circular RNAs, which present this mechanism, are considered miRNA sponges, and they are part of a complex interaction network in the transcriptome or also called the theory of competitive endogenous RNAs(ceRNAs) (Rincon-Riveros et al., 2021).

LINC00968 promotes osteogenic differentiation of DPSCs via regulation of miR-3658/RUNX2 (Liao et al., 2020). LncRNA FER1L4 promotes osteogenic differentiation of human PDLSCs via targeting miR-874-3p (Huang et al., 2020). LncRNA X-Inactive Specific Transcript (XIST) promotes osteogenic differentiation of PDLSCs by sponging miR-214-3p (Feng et al., 2020).

Moreover, circAKT3 positively regulates osteogenic differentiation of human DPSCs via miR-206/CX43 axis (Zhang B. et al., 2020). CircRNA hsa_circ_0026827 has been reported to promote osteoblast differentiation of human DPSCs via Beclin1 and the RUNX1 signaling pathways by sponging miR-188-3p (Ji et al., 2020b). Furthermore, role of exosomes in osteogenic differentiation of DPSCs has been investigated and it was found that circLPAR1 adsorbs to hsa-miR-31 to avoid its inhibitory effects on osteogenesis of DPSCs (Xie et al., 2020).

Based on above, it is well established that the common mechanisms of lncRNAs and circRNAs in DMSCs are competently binding with miRNAs to deliver a promoting effect on osteogenic differentiation.

### ncRNA and Signaling

**FIGURE 3:**
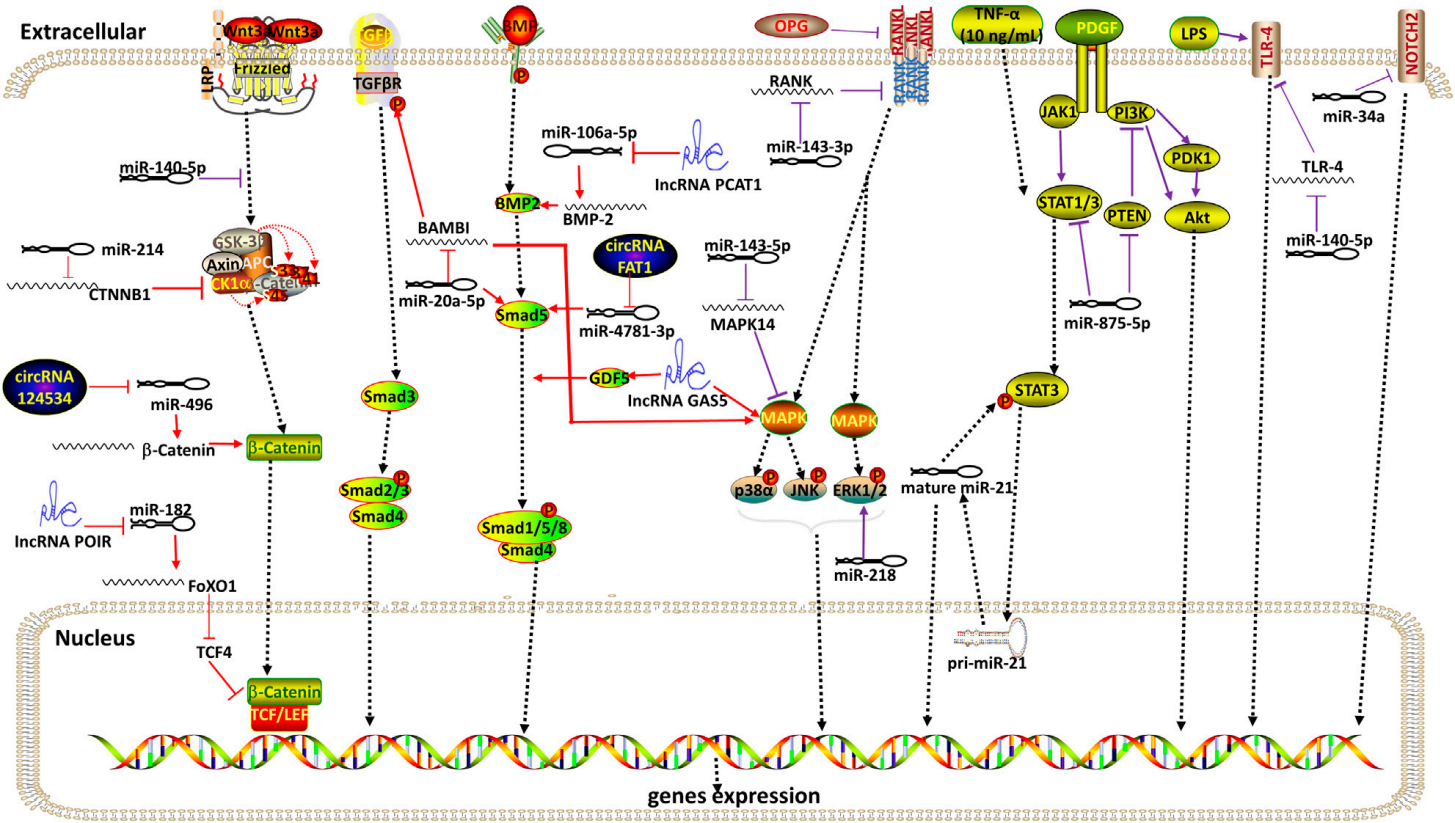
Overview of signaling pathways involved by ncRNAs during the differentiation of DMSCs. Red indicates signaling pathways associated with osteogenic differentiation. Purple indicates signaling pathways associated with odontogenic differentiation. Arrow indicates promotion and T indicates inhibition. Collectively, ncRNAs related to Wnt/β-catenin signaling pathway are miR-140-5p, miR-214, circRNA124534 and lncRNA POIR. MiR-20a-5p is involved in TGF-β, BMP and MAPK signaling pathway. NcRNAs associated with the BMP signaling pathway are lncPCAT1 and circRNA FAT1. LncRNA GAS5 is involved in BMP and MAPK signaling pathway. MiR-143-3p inhibits OPG-RANKL signaling pathway. NcRNAs related to the MAPK pathway are miR-143-5p, miR-218. MiR-875-5P is related to PDGF signaling pathway. There is a reciprocal positive feedback loop between miR-21 and STAT3 at low concentrations of TNF-α (1–10 ng/ml). MiR-140-5p inhibits the TLR-4 pathway under LPS stimulation. MiR-34a inhibits Notch signaling pathway.

From a molecular point of view, DMSCs differentiation is mediated by several signaling pathways. Notably, all three ncRNAs can become key participants in signaling pathways through various interactions with other biomolecules (Figure 3).

### Wnt Signaling Pathway

The Wnt/β-catenin signaling pathway plays an important role in regulating DMSCs differentiation, which is a key signaling pathway. Canonical Wnt signaling is activated by the Wnt1/3a protein when it binds to the Frizzled (FZD), and the low-density LRP5 or LRP6 complex at the cell surface. In the absence of Wnt, β-catenin is phosphorylated by a complex that contains GSK3. β-catenin accumulates in the cytosol and translocates in the nucleus to bind TCF/LEF transcription factor, thereby regulating target gene transcription, such as Runx2, OSX, Hoxc-8 et al. MiR-214 promotes PDLSCs osteoblastic differentiation by directly interacting with the 3′-UTR of the β-catenin gene CTNNB1, and suppressing Wnt/β-catenin signaling through the inhibition of β-catenin (Cao et al., 2017). MiR-140-5p regulates the odontoblastic differentiation of DPSCs *via* the Wnt1/β-catenin signaling pathway (Lu et al., 2019). LncRNA-POIR and miR-182 suppress each other and form a network to regulate FoxO1. FoxO1 increased bone formation of pPDLSCs by competing with TCF-4 for β-catenin and inhibiting the canonical Wnt pathway (Wang et al., 2016). CircRNA124534, acting as a miRNA sponge, directly interacts with miR-496 and consequently regulates β-catenin, which in turn exerts osteogenesis of DPSCs (Ji et al., 2020a).

### MAPK Signaling Pathway

Mitogen-activated protein kinases (MAPKs) are a family of evolutionarily conserved serine/threonine kinases which have three main subfamilies, namely, extracellular signal-regulated kinase (ERK), c jun N-terminal kinase (JNK), and p38. Downregulation of miR-143-5p induces the expression of MAPK14 and odontoblastic differentiation markers (ALP, DSPP, and OCN), which in turn promotes odontogenic differentiation of hDPSCs by activating the p38 MAPK signaling pathway (Wang et al., 2019). MiR-218 plays a negative role in the odontogenesis of DPSCs by promoting ERK1/2 pathway (Chang et al., 2019).

### TGF-β Signaling Pathway

The TGF-β superfamily consists of over 40 members, of which the best known are TGF-βs and Bone morphogenetic proteins (BMPs). In the Smad-dependent signaling pathway, Smad1, Smad5, and Smad8 are BMP activated, whereas Smad 2 and 3 are TGF-β activated. R-Smads form a complex with common-mediator Smad (Co-Smad), Smad4, and enter the nucleus to modulate osteogenic genes expression. Activin membrane binding inhibitor (BAMBI), a TGF-β pseudoreceptor, could interact with TGF-β receptors to inhibit the formation of receptor complexes and then prevent downstream signaling (Onichtchouk et al., 1999). MiR-20a-5p contributes to osteogenic differentiation of hDPSCs through regulating BAMBI and activating the phosphorylation of Smad5 and p38 (Cen et al., 2021). Growth differentiation factor 5 (GDF5) is part of the BMP family. lncRNA growth arrest specific transcript 5 (GAS5) promotes osteogenic differentiation of hPDLSCs by regulating GDF5 and p38/JNK Signaling Pathway (Yang et al., 2020). lncRNA prostate cancer-associated ncRNA transcript-1 (lncPCAT1) promotes the osteogenic differentiation of PDLSCs by sponging miR-106a-5p to upregulate miR-106a-5p-targeted gene BMP2 which activates BMP signaling pathway (Jia et al., 2019). CircRNA FAT1 promotes osteoblastic differentiation of PDLSCs through miR-4781-3p/SMAD5 pathway (Ye et al., 2021).

### RANKL/RANK/OPG Signaling Pathway

RANKL/RANK/OPG system consists of three essential signaling molecules: the cytokine receptor activator of nuclear factor (NF)-kB-ligand (RANKL), the receptor activator of NF-kB (RANK), and the soluble decoy receptor osteoprotegerin (OPG). Binding between RANKL and RANK induces receptor trimerization, which triggers the activation of downstream signaling pathways (such as NF-κB, and the MAPK). (Yang et al., 2020) indicated that down-regulated miR-143-3p resulted in up-regulated RANK and activation of the OPG–RANKL signalling pathway to promote odontoblast differentiation of hDPSCs.

### PDGF Signaling Pathway

Platelet-derived growth factor (PDGF) activates multiple signaling pathways including PI3-kinase pathways. Signal transducer and activator of transcription (STAT) pathway has been shown to interact with PI3-kinase pathways and STATs are regulated by PDGF signaling (Simon et al., 2000). Funada et al. (2020) transfected mimic miR875-5p into mouse DPSCs and found that cell migration toward dental epithelial cells was significantly induced by miR875-5p via the PDGF signaling pathway. Xu et al. (2018) observed that miR-21 promotes the odontogenic differentiation of DPSCs in low concentration (1–10 ng/ml) of TNF-α for that miR-21 and STAT3 have a positive reciprocal feedback loop.

### Notch Signaling Pathway

The Notch signaling pathway is critical for development and cell differentiation. Notch signaling has been confirmed to inhibit odontoblastic differentiation of hDPSCs. (Sun et al., 2014) found that crosstalk between miR-34a and Notch signaling promotes odontoblastic differentiation in SCAPs.

### TLR-4 Signaling Pathway

A certain concentration of Lipopolysaccharides (LPS) promotes the odontoblastic differentiation of DPSCs through the Toll-like receptor (TLR-4) signaling pathway (Zhou et al., 2015). A study (Sun et al., 2017) showed that the expression of miR-140-5p is decreased during LPS-induced odontoblastic differentiation of DPSCs *in vitro* and miR-140-5p suppresses the odontogenic differentiation of DPSCs by binding to the 3′UTR of TLR-4 mRNA.

## Types and Mechanisms of ncRNAs in the Differentiation of DMSCs

Various ncRNAs can affect the differentiation of DMSCs through multiple molecular pathways. The various molecular mechanisms through which ncRNAs regulate the differentiation of DMSC are summarized below.

### Let-7 Family

MicroRNA let-7 family acts as the key regulator of the differentiation of DMSCs. Ma et al. (2016) found that the Insulin-like growth factor-1 (IGF-1)/IGF-1 receptor (IGF-1R)/hsa-let-7c axis can control the odonto/osteogenic differentiation of IGF-1-treated SCAPs via the regulation of JNK and p38 MAPK signaling pathways. Liu G.-X. et al. (2018) demonstrated that overexpression of let-7c significantly inhibited the expression of IGF-1R and downregulated the osteo/odontogenic differentiation of DPSCs. Furthermore, the ERK, JNK, and P38 MAPK pathways were significantly inhibited following the overexpression of hsa-let-7c in DPSCs. *Hsa-let-7b, another member of the let-7 family*, can repress the osteogenic differentiation of PDLSCs by regulating CTHRC1 (Fu et al., 2021). Hsa-let-7b also suppresses the odonto/osteogenic differentiation capacity of SCAPs by targeting matrix metalloproteinase 1 (MMP1) (Wang et al., 2018). As mentioned above, the let-7 family may be an inhibitor of odonto/osteogenic differentiation of DMSCs.

### MiR-17∼92a Family 

**FIGURE 4:**
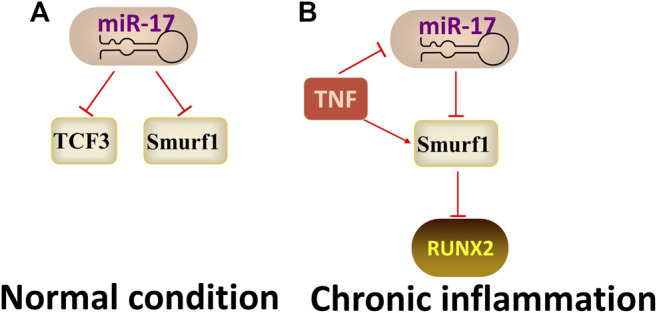
The regulatory mechanism of miR-17. Arrow indicates promotion and T indicates inhibition. **(A)** miR-17 inhibits osteogenic differentiation by targeting TCF3 or Smurf1 when under normal condition. **(B)** miR-17 promotes osteogenic differentiation by binding to Smurf1 when under the TNF-α-induced inflammatory microenvironment.

MiR17-92a family was first described in mammals in 2001, and miR-17 is a core member of the miR-17-92 family. (Liu et al., 2013) found that miR-17 regulated the osteogenic differentiation of PDLSCs by reducing the expression of transcriptional factor 3 (TCF3) and inhibiting the Wnt signalling pathway. In contrast, (Liu et al., 2011) demonstrated that Under normal conditions, miR-17 negatively regulates osteogenic differentiation of PDLSCs, but in the TNF-α-induced chronic inflammatory microenvironment, miR-17 induces osteogenic differentiation by binding to the Smad ubiquitin regulatory factor one (Smurf1, an inhibitor of osteoblast differentiation)3′-UTR. Totally, the function of miR-17 changed depending on the microenvironment (Figure 4).

### MiR-21 

**FIGURE 5:**
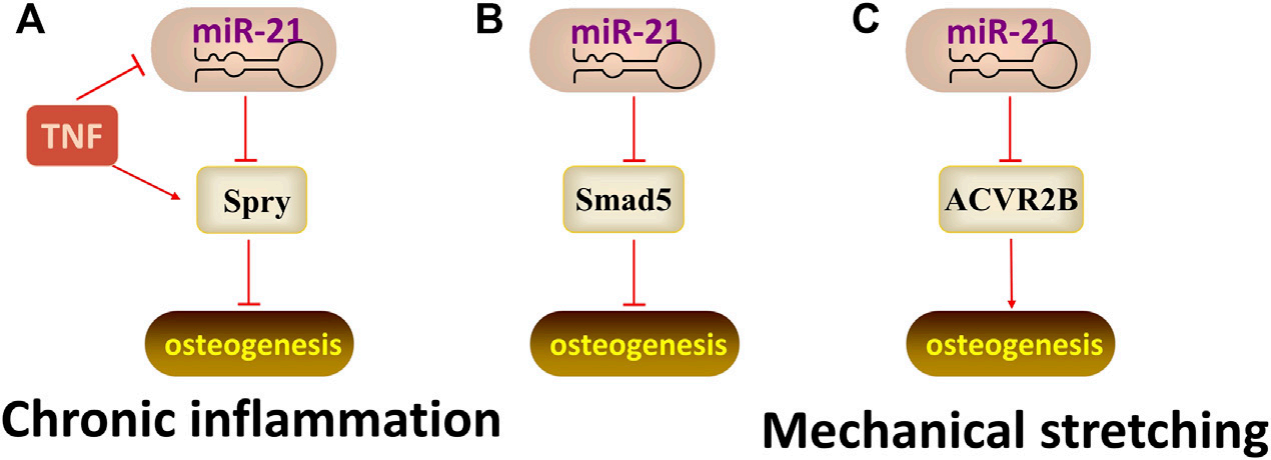
The regulatory mechanism of miR-21. Arrow indicates promotion and T indicates inhibition. **(A)** MiR-21 inhibits osteogenic differentiation by targeting Spry1 when under the TNF-α-induced inflammatory microenvironment. **(B)** MiR-21 inhibits osteogenic differentiation by targeting Smad5. **(C)** MiR-21 promotes osteogenic differentiation by targeting ACVR2B when exposed to mechanical stretch.

The function of miR-21 also influenced by the microenvironment. A study (Yang et al., 2017) showed that the expression of miR-21 was up-regulated during the osteogenic differentiation of PDLSCs Under normal conditions, while the expression of miR-21 was down-regulated in the TNF-α-induced inflammatory microenvironment. Meanwhile, TNF-α suppresses osteogenic differentiation of human PDLSCs by inhibiting miR-21/Spry1 functional axis. In yet another study, miR-21 inhibits the osteogenic differentiation of PDLSCs by targeting the Smad5 (Wei et al., 2017). Interestingly, when exposed to mechanical stretch, miR-21 can target activin receptor type IIB (ACVR2B, a negative regulator of osteogenic differentiation, involved in the TGF-β pathway) to promote the osteogenic differentiation of PDLSCs (Wei et al., 2015). Therefore, miR-21 is a mechanosensitive gene that functions through self-maintenance under different physiological and pathological conditions (Figure 5).

### IncRNA H19

**FIGURE 6:**
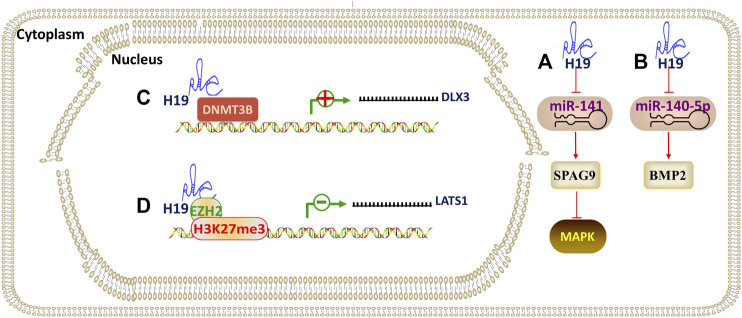
The regulatory mechanism of lncRNA H19. Arrow indicates promotion and T indicates inhibition. **(A)** H19 promotes osteo/odontogenic differentiation through H19/miR-141/SPAG9/MAPK axis. **(B)** H19 promotes odontoblastic differentiation through H19/miR-140-5p/BMP-2/FGF9 axis. **(C)** H19 epigenetically promotes odontoblastic differentiation by inhibiting DNMT3B-mediated methylation of DLX3. **(D)** H19 epigenetically promotes odontoblastic differentiation by repressing LATS1 through EZH2-induced trimethylation of H327me3.

The lncRNA H19 is transcribed from the H19/IGF2 gene located on human chromosome 11p15.5 and has a molecular weight of 2.3 kilobase. Recent studies have shown that H19 can participate in the differentiation of DMSCs by adsorbing and inhibiting the expression of miRNAs (acting as a ceRNA). (Li et al., 2019) revealed that H19 promotes the osteo/odontogenic differentiation of SCAPs and “H19/miR-141/SPAG9/MAPK” positive feedback loop plays paramount role. Mechanistically, H19, as a ceRNA, serves as a miRNA sponge for miR-141. H19 competitively bound to miR-141 and prevented SPAG9 from miRNA-mediated degradation, thus significantly elevating phosphorylated levels of p38 and JNK and facilitating the osteo/odontogenic differentiation of SCAPs. Zhong et al. (2020) found that H19 plays a positive regulatory role in odontoblastic differentiation of hDPSCs through H19/miR-140-5p/BMP-2/FGF9 axis. H19 is both epigenetically regulated and utilizes epigenetic mechanisms to regulate odontogenic differentiation of hDPSCs. Zeng et al. (2018) demonstrated that the overexpression of H19 reduced the expression level of S-adenosylhomocysteine hydrolase (SAHH), which can block the methylation activity of DNA methyltransferases (DNMTs). Besides, upregulated H19 expression significantly repressed SAHH and DNMT3B activity, which then enhanced the DLX3 expression by inhibiting DNMT3B-mediated methylation of DLX3. Therefore, the H19/SAHH axis epigenetically promoted odontogenic differentiation of hDPSCs. (Du et al., 2021) revealed that H19 inhibited LATS1 to promote the production of odontoblasts. The effects of H19 on hDPSCs were achieved by repressing LATS1 through EZH2-induced trimethylation of H327me3 (Figure 6).

### IncRNA ANCR

Anti-differentiation non-coding RNA (ANCR, subsequently named differentiation-antagonizing non-protein coding RNA (DANCR)), was identified in 2012, which function was mainly to inhibit differentiation and to enhance the undifferentiated state of somatic progenitor populations (Kretz et al., 2012). lncRNA DANCR suppresses odontoblast-like differentiation of human DPSCs by inhibiting wnt/β-catenin pathway (Chen et al., 2016). (Jia et al., 2015) found that down-regulated lncRNA-ANCR promoted osteogenic differentiation of PDLSCs and the regulating effect of lncRNA-ANCR was associated with the canonical WNT signalling pathway. Peng et al. (2018) showed that lncRNA-ANCR suppresses bone formation of PDLSCs via sponging miRNA-758. Furthermore, downregulation of lncRNA-ANCR promoted the osteogenic, adipogenic and neurogenic differentiation of DMSCs (DPSCs, PDLSCs and SCAPs Jia et al., 2016). These data provide an insight effect of lncRNA-ANCR on DMSCs and indicate that ANCR is a vital regulatory factor in DMSCs differentiation.

### lncRNA MEG3

**FIGURE 7:**
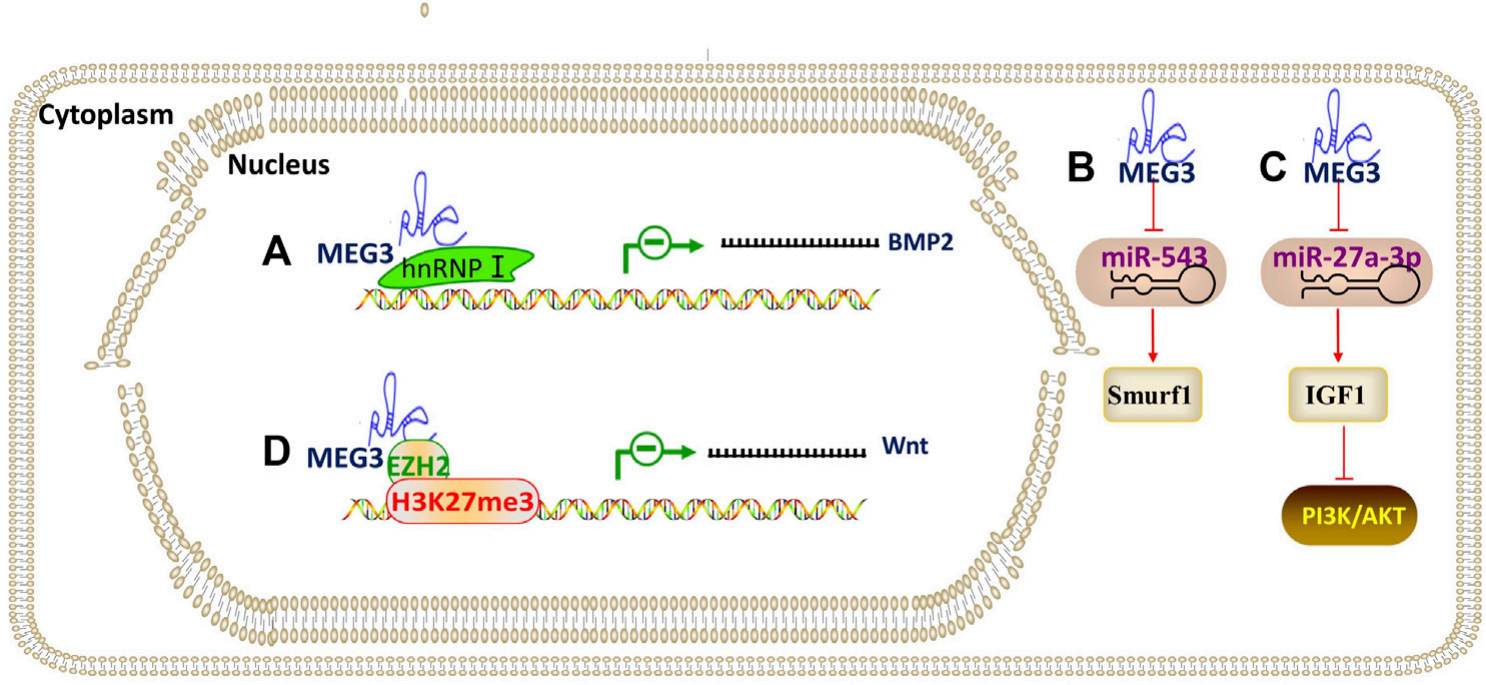
The regulatory mechanism of lncRNA MEG3. Arrow indicates promotion and T indicates inhibition. **(A)** MEG3 leads to repression of BMP2 by competing with BMP2 mRNA for hnRNPs (an RNA-binding protein), which in turn inhibits osteogenic differentiation. **(B)** MEG3 promotes osteogenic differentiation through MEG3/miR-543/Smurf1 axis. **(C)** MEG3 promotes osteogenic differentiation through MEG3/miR-27a-3p/IGF1/PI3K/Akt axis. **(D)** MEG3 epigenetically regulating Wnt through the EZH2 regulation of H3K27me3 level on the Wnt genes promotors.

Maternally expressed gene 3 (MEG3), also known as gene trap locus 2 (Gtl2), has been considered as a lncRNA for tumor suppression. MEG3 is known to hinder osteogenic differentiation of PDLSCs by competing with BMP2 mRNA for heterogeneous nuclear ribonucleoprotein I (hnRNPs, an RNA-binding protein), leading to repression of BMP2 (Liu et al., 2019b). Another study (Zhao et al., 2020) demonstrated that MEG3 increases the Smurf1 by serving as a ceRNA to sequester miR-543 in human DPSCs. MEG3 also promotes the osteogenic differentiation of PDLSCs by targeting miR-27a-3p for the upregulation of IGF1 and activation of PI3K/Akt signaling (Liu et al., 2019a). Downregulation of MEG3 resulted in enhancement of osteogenic differentiation of human DFPCs through epigenetically regulating the Wnt/β-catenin pathway, and ChIP analysis showed that these effects were due to the EZH2 regulation of H3K27me3 level on the Wnt genes promotors (Deng et al., 2018). As mentioned above, MEG3 coordinates osteogenic differentiation in DMSCs by targeting multiple molecules (Figure 7).

### CircRNA CDR1as

Antisense to the cerebellar degeneration-related protein 1 transcript (CDR1as), also known as CIRS-7, has approximately 70 conserved miR-7 binding sites and could therefore act as a miR-7 “sponge”. (Gu et al., 2021) demonstrated that CDR1as promotes the pluripotent state of PDLSCs by inhibiting miR-7-mediated suppression of KLF4 expression, and hnRNPM can promote the expression of CDR1as in PDLSCs. CDR1as was also reported (Li X. et al., 2018) to regulate the osteoblastic differentiation of PDLSCs by triggering the activation of Smad and p38 MAPK signaling pathway, as well as upregulation of GDF5. The circRNA-CDR1as/miR-7/GDF5/SMAD and p38 MAPK signaling pathway may be involved in PDLSCs upon the periodontitis burst.

### CircRNA SIPA1L1

CircSIPA1L1 is produced by a transcript encoding circSIPA1L1 on human chromosome 14. The expression of circSIPA1L1 in the mineralization induction group was about 8 times that of the control group, suggesting vital functions of circSIPA1L1 in osteogenesis. CircSIPA1L1 was found to regulate miR-617/Smad3 axis to potentiate osteogenic differentiation of DPSCs (Ge et al., 2020). In yet another study, circSIPA1L1 regulated osteoblastic differentiation of SCAPs via miR-204-5p/ALPL pathway (Li Y. et al., 2020).

## Conclusion

In this review, we summarized the functions and mechanisms of ncRNAs, which played important roles in the differentiation of DMSCs. DMSCs are currently considered as valid biological sources for use in tissue regeneration by tissue engineering approaches. Therefore, deep knowledge of the mechanisms which govern DMSCs differentiation is of pivotal relevance. During the differentiation of DMSCs, all three ncRNAs are regulated by multiple signalling pathways. MiRNAs regulate gene expression by targeting the 3′UTR of target genes, while they are influenced by the epigenetics and microenvironment (inflammation, mechanical stretching), forming a complex and intertwined regulatory network. LncRNAs have broad functional versatility, because they contribute to the regulation of gene expression at different levels in the nucleus or cytoplasm and at transcriptional and post-transcriptional levels. Both circRNAs and lncRNAs function as ceRNAs, and there may be a competitive lncRNA/circRNA-miRNA-mRNA regulatory network system. With increasing numbers of miRNAs, lncRNAs and circRNAs discovered in this process, it has become possible to use these ncRNA-related therapeutic methods in the field of tissue repair and regeneration.

However, the current studies on the regulation of multi-directional differentiation of DMSCs by ncRNAs mostly focus on the following aspects: odontogenic differentiation, osteogenic differentiation, neurogenic differentiation, angiogenic differentiation and myogenic differentiation. DMSCs also have the ability to differentiate into liver tissue-like cells and islet-like cells, and reports on the regulation of ncRNAs in these tissue-like cells are still lacking. Therefore, further studies on the differentiation of other tissue-like cells are encouraged. Moreover, the related markers, signaling pathways (Wnt, TGFβ, MAPK), and ncRNAs (hsa-let-7c, H19) partially overlap during the osteogenic and odontogenic differentiation of DMSCs. There should be more reports in the future explaining the difference and connection of ncRNAs in their differentiation. Besides, most studies are primarily performed *in vitro*, and such *in vitro* differentiation is often limited and may not represent true differentiation of the cells themselves. Therefore, further studies on ncRNAs are needed, including *in vivo* experiments and animal disease models. Finally, different types of DMSCs have the ability to differentiate into different lineages. Among all subpopulations of DMSCs, DPSCs and PDLSCs are most extensively studied. Future studies should emphasize the effective combination of ncRNAs with various types of DMSCs to provide a potential scheme for tissue regeneration. Overall, whereas interest and investigation in the contribution of ncRNAs to the differentiation of DMSCs have increased considerably, the field is still a long way from understanding the full extent of the contribution of ncRNAs and the mechanisms by which ncRNAs exert their potential effects in this field.
